# Reactive oxygen and nitrogen (ROS and RNS) species generation and cell death in tomato suspension cultures—*Botrytis cinerea* interaction

**DOI:** 10.1007/s00709-014-0680-6

**Published:** 2014-07-27

**Authors:** E. Pietrowska, S. Różalska, A. Kaźmierczak, J. Nawrocka, U. Małolepsza

**Affiliations:** 1 Department of Plant Physiology and Biochemistry, University of Lodz, Banacha 12/16, 90-237 Lodz, Poland; 2 Department of Industrial Microbiology and Biotechnology, University of Lodz, Banacha 12/16, 90-237 Lodz, Poland; 3 Department of Cytophysiology, University of Lodz, Banacha 12/16, 90-237 Lodz, Poland

**Keywords:** *B. cinerea*, Cell death, Nitric oxide, Resistance, Tomato

## Abstract

This article reports events connected to cell survival and *Botrytis cinerea* infection development in cell suspension cultures of two tomato cultivars which show different levels of susceptibility to the pathogen: cv. Corindo (more susceptible) and cv. Perkoz (less susceptible). In parallel changes in reactive oxygen (ROS) and nitrogen (RNS) species generation and in *S*-nitrosoglutathione reductase (GSNOR) activity were studied. In vivo staining methods with acridine orange (AO) and ethidium bromide (EB) as well as fluorescent microscopy were used to assess tomato and *B. cinerea* cells death. The biochemical studies of ROS and RNS concentrations in plant cell extract were complemented by in vivo ROS and nitric oxide (NO) imaging using nitro blue tetrazolium (NBT), diaminobenzidine (DAB) and diaminofluorescein diacetate (DAF-DA) staining methods, and confocal microscope technique. *B. cinerea* infection proceeded slower in Perkoz cell cultures. It was evidenced by measuring the pathogen conidia germination and germination tube development in which nuclei revealing cell death dominated. Two different types of tomato cell death were observed: cells with necrotic nuclei dominated in Corindo whereas in Perkoz cells with characteristic of vacuolar death type prevailed. In Perkoz cells, constitutive levels of NO and *S*-nitrosothiols (SNO) were significantly higher and hydrogen peroxide (H_2_O_2_) and superoxide anion (O_2_^−^) concentrations were slightly higher as compared with Corindo cells. Moreover, increases in these molecule concentrations as a result of *B. cinerea* inoculation were observed in both, Perkoz and Corindo cell cultures. The enzymatic GSNOR activity seems to be an important player in controlling the SNO level in tomato cells. Involvements of the studied compounds in molecular mechanisms of tomato resistance to *B. cinerea* are discussed in the paper.

## Introduction

The necrotrophic pathogen *Botrytis cinerea* is a casual agent of gray mold in a broad host range (Elad et al. 2007; Finkers et al. 2007). It is one of the most devastating diseases of tomato grown in field and glasshouse conditions. The pathogen infects leaves, stems, flowers, and tomato fruits during cultivation as well as during transport and storage. Modern hybrid tomato cultivars are susceptible to *B. cinerea*. Although some cultivars show some level of resistance, commercially acceptable resistant tomato cultivars are not available so far.

*B. cinerea* is difficult to control because it has a variety of modes of attack, diverse hosts as inoculum source, and it can survive as mycelia and/or conidia for extended periods as sclerotia in crop debris. For these reasons, the use of any single control measure is unlikely to succeed and more detailed understanding of the biochemical bases of this host-pathogen interaction is essential (Williamson et al. 2007). Plant defense mechanisms against necrotrophs, such as *B. cinerea*, are complex, and despite extensive studies, their biochemical bases are still not fully understood and are a matter of controversial debate (Asai and Yoshioka 2009; Asselbergh et al. 2007; Floryszak-Wieczorek et al. 2007; Govrin and Levine 2000; Oirdi et al. 2011; Unger et al. 2005).

An oxidative burst, a common early response of host plant cells to pathogen attack and elicitor treatment, is one of the crucial components of plant defense responses (Delledonne et al. 2001; Zaninotto et al. 2006). Studies of various plant-pathogen combinations have revealed a striking correlation between the profile of ROS formation and the outcome of the interaction (resistance or susceptibility) in plants. ROS, predominantly O_2_^.-^ and H_2_O_2_, overproduced during the oxidative burst may be directly involved in pathogen killing (Peng and Kuć 1992; Wang and Higgins 2005) and strengthening of a plant cell wall as well as in triggering hypersensitive cell death (HR) and in production of systemic resistance signaling (Delledonne et al. 2001; Zaninotto et al. 2006). Death of attacked cells during HR, preceded by oxidative burst, has been considered an important element of successful defense strategy of plants against biotrophic pathogens feeding on living host tissues. Enhanced ROS generation was also found to accompany an infection caused by necrotrophs, but in that case, their role in the interaction is still controversial; death of host cells during HR is considered advantageous for the pathogen. Necrotrophs kill their host cells by secreting toxic compounds or lytic enzymes, and in addition, they produce different pathogenic factors that can subdue host defense. The ability of the fungus to kill cells was proposed as an important determinant in host susceptibility to different *Botrytis* species; plant resistance to the pathogen is supposed to depend on the balance between cell death and survival (van Barleen et al. 2007; Asselbergh et al. 2007). ROS production does not always result in increased susceptibility, because failure or success of infection by *B. cinerea* appears to depend strongly on the timing and the intensity of oxidative burst (Asai and Yoshioka 2009; Asselbergh et al. 2007; Kunz et al. 2006; Shlezinger et al. 2011).

Considerable evidence indicates that ROS generation is accompanied by nitric oxide (NO) synthesis (Asai and Yoshioka 2009; Chaki et al. 2009; Zaninotto et al. 2006). NO and ROS interplay is of special interest in plant disease resistance initiation and execution. Nitric oxide together with ROS have been identified as essential molecules that mediate cell death in HR and defense gene activation (Lin et al. 2012; Zaninotto et al. 2006). It is suggested that *S*- nitrosoglutathione acts as a long-distance signal in systemic acquired resistance (SAR) that might act both as NO reservoir and NO donor (Lindermayr et al. 2005; Restérucci et al. 2007); NO is indispensable to salicylic acid (SA) functioning as SAR inducer (Malik et al. 2011; Romero-Puertas and Delledonne 2003). *S*-nitrosylation, addition of NO moiety to Cys thiol to form *S*-nitrosothiol (SNO), has now emerged as a key redox-based posttranslational modification in plants and a major route for the transduction of NO bioactivity integral to plant immune function (Feechan et al. 2005; Lin et al. 2012; Malik et al. 2011). Growing evidence suggests that NO and SNO are important mediators in the process of plant cell death induction and orchestration (de Pinto et al. 2012; Lin et al. 2012; Malik et al. 2011). Cellular SNO level is regulated by *S*-nitrosoglutathione reductase (GSNOR). The function of this enzyme is conserved between bacteria, animals, and plants (Liu 2001; Lin et al. 2012). GSNOR seems to be an important player in plant de-nitrosylation, especially during development of disease response; however, its precise role in this process is far from being clear (Feechan et al. 2005; Malik et al. 2011; Wang et al. 2009).

Accumulating evidence suggests that both NO and ROS play key roles in programmed cell death (PCD) which is an integral part of plant development and defense. Relatively, little is known about PCD in plants, and detailed mechanisms underlying this still need elucidation. Various types of plant PCD have been proposed (Love et al. 2008; van Doorn et al. 2011; Byczkowska et al. 2013). Two major types of plant cell death have been described: vacuolar cell death and necrosis. Vacuolar cell death is connected with formation of lytic vacuoles, tonoplast rupture, and releasing of hydrolases, gradual decreasing cytoplasm as well as nuclei segmentation and chromatin condensation. This type of PCD mainly occurs during normal plant development and after mild abiotic stress. Necrosis is characterized by early rupture of plasma membrane, shrinking of protoplast, and absence of growing lytic vacuoles. The third type of plant cell death is connected with HR response to pathogens which can express features of both necrosis and vacuolar cell death (van Doorn et al. 2011). HR exhibits different patterns of cellular changes depending on host-pathogen interactions. This type of cell death has been observed during interactions with biotrophic as well as necrotrophic pathogens. HR usually occurs at the site of successful recognition of biotrophic pathogens feeding on living host tissues. Cell death at the site of pathogen attack restricts pathogen invasion and disease development. On the contrary, the ability of the necrotrophic pathogens to kill cells was proposed as a determinant in host susceptibility. However, the data regarding HR cell death contribution to the defense responses associated with necrotrophic pathogens are contradictory.

In the present work, using cell cultures of two tomato cultivars differing in resistance to *B. cinerea*, we undertook a biochemical and cellular study of changes in ROS, NO, SNO, MDA concentrations, and GSNOR activity as well as tomato cell viability and the pathogen infection development to explain the biochemical bases of tomato resistance to the pathogen.

## Material and methods

### Plant material

Cell suspension cultures of two tomato (*Solanum lycopersicum* L.) cultivars: Corindo—more susceptible to *B. cinerea* and Perkoz—less susceptible were grown in Chandler medium supplemented with BAP 0.2 mg dm^−3^ and 2,4 D 1.0 mg dm^−3^ (Chandler et al. 1972). Established cell cultures were subcultured by pipetting 25 cm^3^ of 7-day-old cultures into 75 cm^3^ of fresh growth medium in 300 cm^3^ Erlenmeyer flasks. The subcultured cell cultures were grown in the dark at 25 °C, on a rotating platform at 120 rpm. Three-day-old cultures with cell density 10^6^ cm^−3^ were taken to experiments; some of them were inoculated with 2 cm^3^ *B. cinerea* conidia suspension (10^5^ cm^−3^). Control, noninoculated, and pathogen inoculated cell cultures were harvested and examined 6, 12, 24, and 48 h postinoculation (hpi). The cells were separated from the growth medium using vacuum-assisted filtration through two layers of Miracloth (Calbiochem, San Diego, CA, USA).

### *B. cinerea* culture

*B. cinerea* isolate 1631 was provided by Bank of Plant Pathogens (Poznań, Poland) and was maintained in stock culture on potato dextrose agar in the dark at 24 °C. The conidial suspension was obtained by washing potato dextrose agar slant cultures with tap water. 1 × 10^5^ cm^−3^ conidial suspension was used to inoculate tomato cell cultures.

### Assay of *B. cinerea* infection development in tomato cell cultures

*B. cinerea* infection development in tomato cell cultures was assayed as a percentage of conidia germination. The percentage of germinated *B. cinerea* conidia was determined microscopically 6, 12, 24, and 48 hpi. Conidia were considered germinated when the length of germ tubes exceeded the diameter of the conidium.

### Assay of viability of cell cultures

The Evans blue method was used to test cell viability/death according to Kanai and Edwards (1973) with modification. Briefly, 1 cm^3^ of Evans blue solution (0.25 % Evans blue in 3 mM CaCl_2_ and 0.6 M mannitol) was added to 0.1 g of cells for 10 min. The cells were washed in 2 cm^3^ of water for 30 min. Drops of cell suspension were put on Füsch-Rosenthal camera and analyzed using light microscope. Dead (dark blue) and viable (non-stained) cells were counted in twenty samples for each treatment, with every experiment repeated at least three times.

### Assay of cell death by fluorescent microscopy

Detection and verification of cell death in the suspension of cells were carried out according to the following procedure:

1. Culture medium (0.5 cm^3^) with 0.5 cm^3^ of appropriate cell suspension was supplemented with 0.5 cm^3^ of 0.02 M phosphate buffer pH 7.4 (PHB).
2. The cells were stained with the staining mixture containing 50 μg cm^−3^ AO (acridine orange) and 50 μg cm^−3^ EB (ethidium bromide) at PHB.
3. Drops of cell suspension were immediately put on glass slides and analyzed for 5 min using fluorescent microscopy with a blue light (B2A) excitation filter of the Optiphot-2 epi-fluorescence microscope (Nikon) equipped with a camera and Act-1 software (Precoptic, Poland) for fluorescent microscopy and preparation of microphotographs according to Byczkowska et al. 2013.

AO/EB staining consists in staining with AO which permeates whole cells and makes the nuclei green and with EB which is only taken up by cells when the cellular and nuclear membrane integrity is lost and stains the nuclei red. EB also dominates over AO (Ribble et al. 2005; Kobori et al. 2007). It has been reported (Kobori et al. 2007; Byczkowska et al. 2013) that under fluorescence microscope, living cells have unchanged green nuclei while dying cells have green-yellow, yellow, yellow-orange, and bright orange nuclei with slightly condensed or fragmented chromatin at the early stage of death while at the late one with condensed and fragmented chromatin. Necrotic cells have structurally normal orange nuclei (Ribble et al. 2005; Byczkowska et al. 2013). These cells were described as dead (Byczkowska et al. 2013). When the color is changed from green to red, values of fluorescence intensity of both fluorochromes increase. Thus, according to the values of curve of the resultant fluorescence intensity (Byczkowska et al. 2013) it is possible to assign cells as alive, dying or dead and present their number in appropriate index (Byczkowska et al. 2013).

### Assay of nitro blue tetrazolium reducing activity

Measurement of nitro blue tetrazolium (NBT) (Sigma-Aldrich Chemie GmbH, Steinheim, Germany) reduction, a method used for the determination of O_2_^.-^, was described by Doke (1983). Twenty-five milligrams of cells were resuspended in 3 cm^3^ 0.01 M potassium phosphate buffer pH 7.8 containing 0.05 % NBT and 10 mM NaN_3_ (Sigma-Aldrich) for 1 h. After removing the cells by filtration through paper filter, the mixture was heated at 85 °C for 15 min and cooled. The NBT reducing activity of the cells was expressed as increased absorbance at 580 nm h^−1^ g^−1^ of fresh weight. The effect of SOD on the reduction of NBT by the cells was determined by adding SOD (manganese-containing enzyme) (Sigma-Aldrich) (100 μg ml^−1^) to the reaction solution from which NaN_3_ was omitted.

### Assay of hydrogen peroxide concentration

Hydrogen peroxide was measured by the method described by Capaldi and Taylor (1983) with slight modifications. Two hundred fifty milligrams of cells were ground in 2.5 cm^3^ 5 % TCA with 50 mg active charcoal at 0 °C and centrifuged for 10 min at 15,000*g*. Supernatant was collected, neutralized with 4 N KOH to pH 3.6 and used for H_2_O_2_ assay. The reaction mixture contained 200 μl of cell extract, 100 μl of 3.4 mM 3-methylbenzothiazoline hydrazone (MBTH, Sigma-Aldrich). The reaction was initiated by adding 500 μl of horseradish peroxidase (Fluka Chemie GmbH) solution (90 U per 100 cm^3^) in 0.2 M sodium acetate buffer pH 3.6. Two minutes later, 1,400 μl of 1 N HCl was added. Absorbance was determined at 630 nm. H_2_O_2_ concentration was calculated based on a standard curve of the H_2_O_2_ and expressed in μmol g ^−1^ fresh weight.

### Assay of nitric oxide concentration

Nitric oxide content was determined using the method described by Ding et al. (1988) with slight modifications. Six hundred milligrams of cells were ground in 3 cm^3^ of 50 mM cool acetic acid buffer pH 3.6 containing 4 % zinc diacetate. The homogenate was centrifuged at 10,000*g* for 15 min at 4 °C. The supernatant was collected. The pellet was washed by 1 cm^3^ extraction buffer and centrifuged as before. The two supernatants were combined and 0.1 g of charcoal was added. After vortex and filtration, the filtrate was leached and collected. The mixture of 1 cm^3^ of filtrate and 1 cm^3^ of the Griess reagent (Sigma-Aldrich) was incubated at room temperature for 30 min. Absorbance was determined at 540 nm. NO content was calculated by comparison to a standard curve of NaNO_2._

### Assay of in situ NO accumulation

Five microliter of 10 mM stock solution of 4,5-diaminofluorescein diacetate (DAF-2DA) in DMSO (Sigma-Aldrich)—the probe used for visualization of NO, was added to 2.5 cm^3^ of cell suspension culture. After 30-min incubation at 25 °C in the dark, the cells were filtered and washed with 10 mM Tris-HCl buffer pH 7.0, mounted in the buffer on microscope slides, and then examined immediately under confocal laser scanning microscope Pascal 5 (Zeiss). The cells were excited with the 488 line of an argon laser. The emission was recorded using a 530-nm bandpass filter. For Nomarski DIC the same laser line as described above was used. Constant exposure time was used for all experiments. The production of green fluorescence under these conditions was attributed to the presence of NO (Foissner et al. 2000). The slides were scanned into a computer. Microscope, laser, and photo multiplier settings were held constant during the course of all experiments in order to obtain comparable results from observation of at least triplicate samples from each experiment, with every experiment repeated at least three times.

### Assay of SNO concentration

Total SNO levels were determined according to Restérucci et al. 2007. Proteins were extracted in 100 mM Tris-HCl, pH 6.8. The extracts were incubated for 5 min with an equivalent volume of solution A (1 % sulfanilamide dissolved in 0.5 M HCl) in the presence or absence of solution B (solution A plus 0.2 % HgCl_2_), allowing the development of the diazonium salt. The formation of the azo dye product was obtained by reacting the two samples for an additional 5 min with an equal volume of solution C [0.02 % of N-(1-naphthyl) ethylenediamine dihydrochloride dissolved in 0.5 M HCl], and the absorbance was subsequently read at 550 nm with spectrophotometer (Hitachi, F-2500). SNO was quantified as the difference of absorbance between solution B and A (B-A), comparing the values with a standard curve made from solution of GSNO (Sigma-Aldrich). The results were normalized per milligram of protein measured by the Bradford (1976) method.

### Assay of in situ accumulation of O_2_^.−^ and H_2_O_2_

In situ generation of O_2_^.-^, was visualized using NBT forming dark blue insoluble precipitate in the presence of O_2_^.-^. Such reaction was not observed in the presence of SOD (data not shown). A modified method described by Trujillo et al. (2004) was used. The control and *B. cinerea* inoculated cells were collected at 12 hpi and incubated with 0.1 % solution of NBT in 10 mM phosphate buffer pH 7.8 containing 10 nM Na-azide for 3 h in the light and at room temperature.

In situ generation of H_2_O_2_ was detected by formation of brown precipitate after incubation of the cells with a solution of 1 mg cm^−3^ 3,3′-diaminobenzidine-tetrahydrochloride (DAB) (Sigma-Aldrich) pH 3.8 for 8 h in the light and at room temperature according to the modified method of Orozco-Cárdenas and Ryan (1999). The cells were preserved in cool ethanol and photographed with Axiovert 200 M inverted microscope equipped with HRC digital camera (Zeiss).

### Assay of lipid peroxidation (MDA content)

Lipid peroxidation was determined by measuring the concentration of thiobarbituric acid-reactive substances (TBARS)—MDA content according to Yagi (1976) with modifications. The biomass of 0.5 g of cells was mechanically homogenized (1:10 *w*/*v*) with a 50 mM sodium phosphate buffer pH 7.0 containing 1 N NaCl, 1 % PVP (Sigma-Aldrich) MW 40,000, and 1 mM ascorbate (Sigma-Aldrich) at 4 °C. After centrifugation at 15,000*g* for 15 min, the supernatant was collected. The supernatant was mixed with TBA solution (1:1 *v*/*v*) and heated at 95 °C for 1 h. After cooling, the samples were supplied with *n*-butanol and intensively shaken. After centrifugation (10,000*g*, 10 min), the obtained organic layer was separated and its fluorescence was measured at 531 nm (excitation) and 553 nm (emission) with fluorescence spectrometer. The concentration of MDA was estimated by referring to a standard 1,1,3,3-tetraetoxypropane and expressed as nM of MDA calculated per 1 g fw.

### Preparation of enzyme extracts

At 4 °C, 0.25 g of cells was homogenized in 2.50 cm^3^of 50 mM Tris-HCl buffer pH 8.0 containing 0.5 M NaCl. After centrifugation at 15,000*g* for 15 min, the supernatant was collected.

### Assay of *S*-nitrosoglutathione reductase (GSNOR) activity

GSNOR activity was determined by the modified method of Sakamoto et al. (2002). Enzyme activity was measured spectrophotometrically at 25 °C by monitoring the decomposition of NADH at 340 nm. The reaction mixture contained 20 mM Tris-HCl buffer pH 8.0, 0.2 mM NADH, 0.5 mM EDTA, and GSNO with final concentration 400 μM. GSNOR enzyme activity was calculated taking into account the millimolar extinction coefficient of NADH ε = 6.22 mM cm^−1^ at 340 nm and presented in NADH min^−1^ mg^−1^ of protein.

### Assay of protein content

Protein was determined by the method of Bradford (1976) with standard curves prepared using bovine serum albumin (Sigma-Aldrich).

### Statistical analysis

The significance of differences between mean values obtained from four independent experiments with three replicates each was determined by Student’s-test. Sample variability is given as standard deviation (S.D.). Differences at *p* < 0.05 were considered significant.

## Results

### *B. cinerea* infection development and plant cell viability in tomato cell cultures

**Fig. 1:**
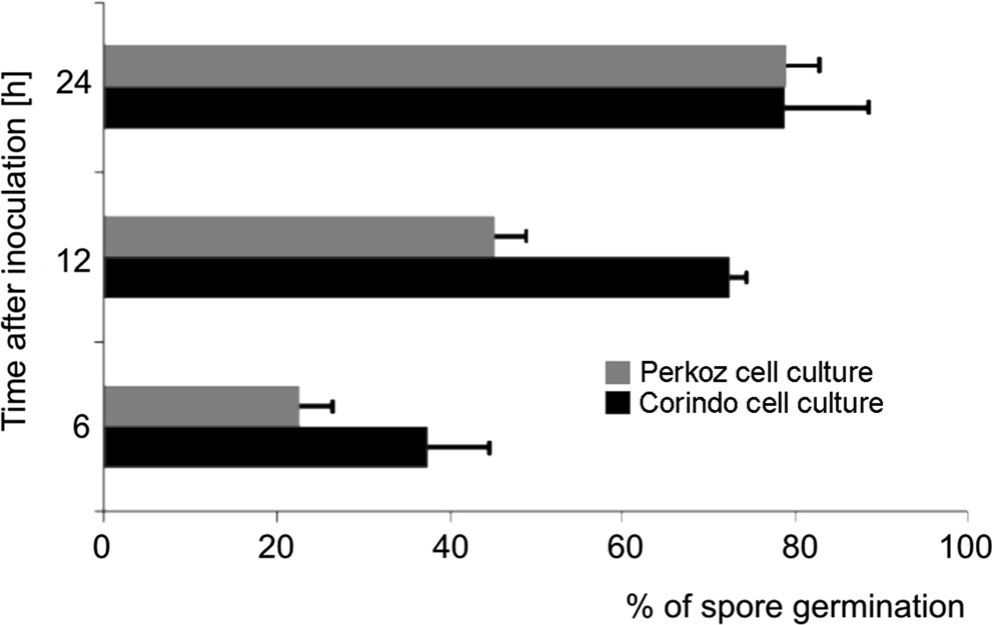
*B. cinerea* conidia germination in tomato cell cultures. *Values* represent the means and S.D. from four independent experiments with three replicates each, *n* = 12

**Fig. 2:**
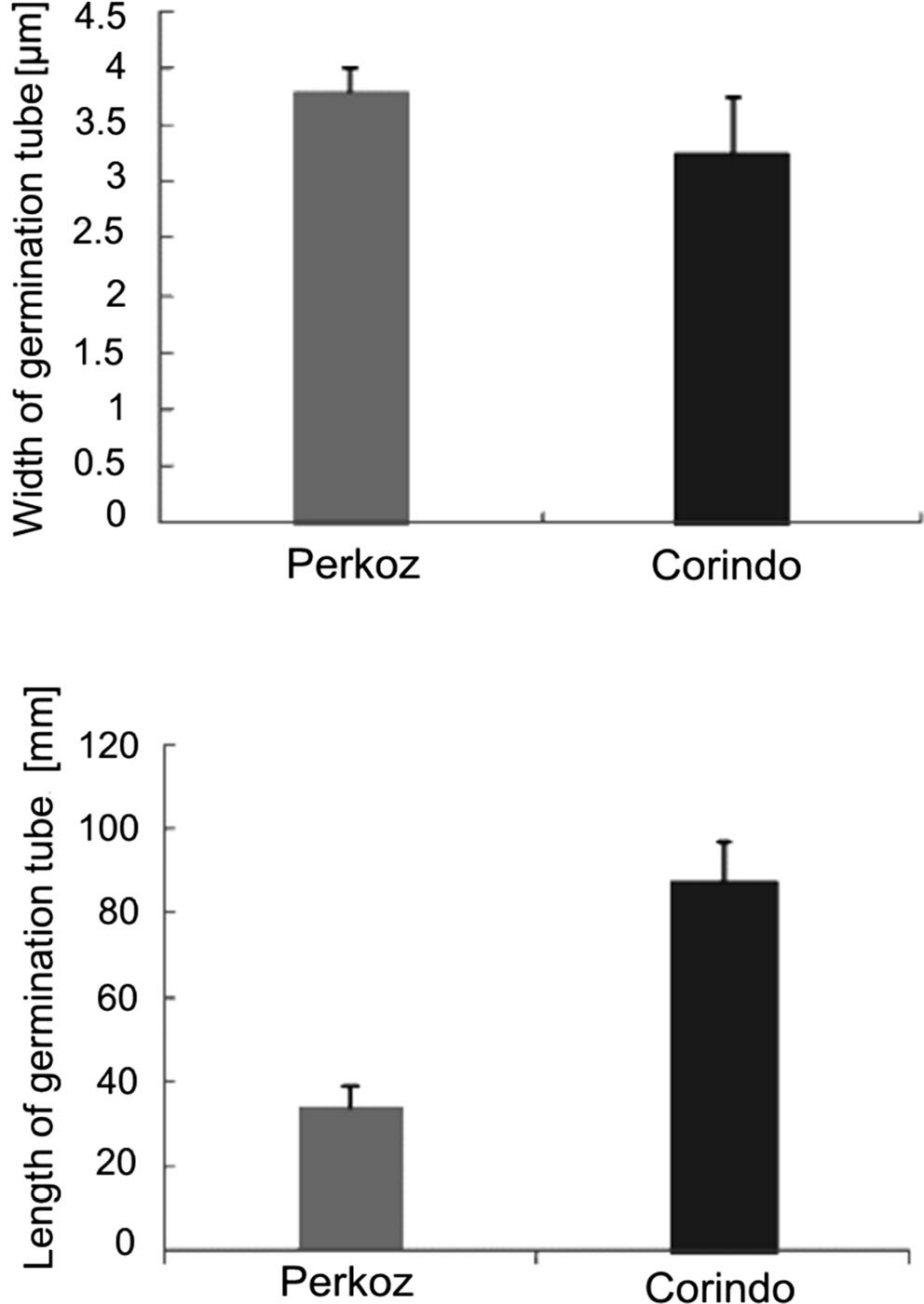
*B. cinerea* germination tube development in tomato cell cultures at 12 hpi. Data and statistic as in Fig. 1

**Fig. 3:**
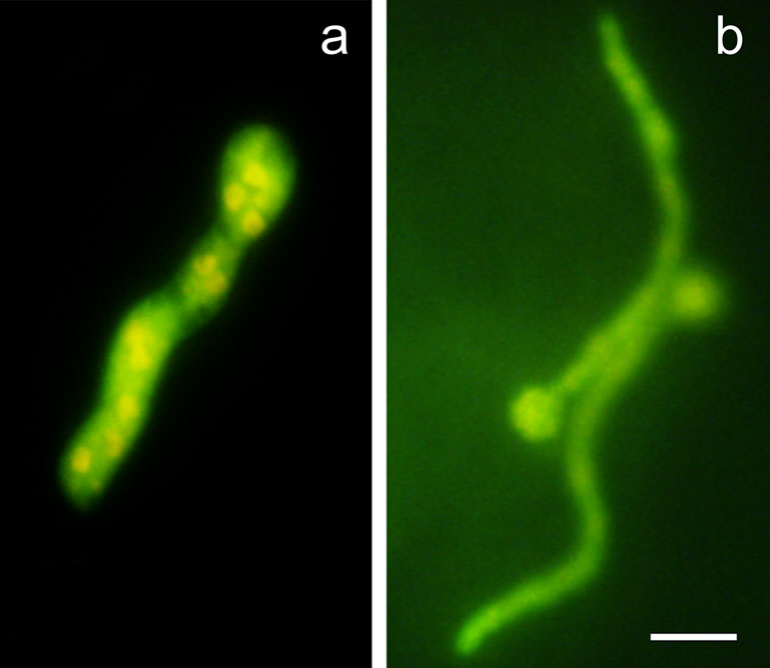
*Yellow*-*orange* nuclei in cells dying via vacuolar type of death of *B. cinerea* germination tubes in Perkoz cell culture (**a**) and *green* nuclei in living cells of *B. cinerea* germination tubes in Corindo cell culture (**b**) at 12 hpi, detected by staining by AO/EB. *Bar*: 20 μm is applied to (**a**) and (**b**)

**Fig. 4:**
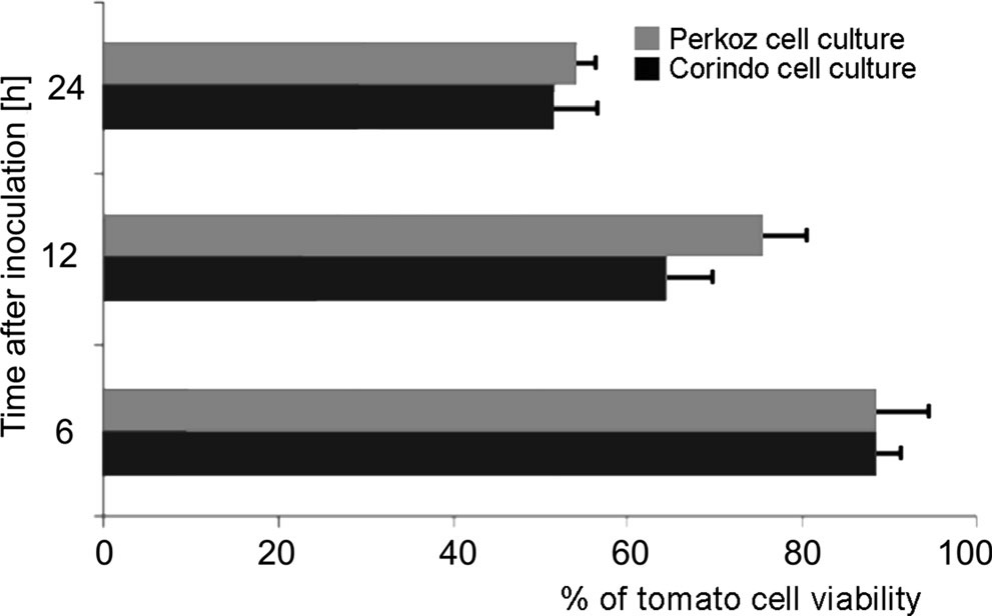
Cell viability in tomato cell cultures inoculated with *B. cinerea*. Data and statistic as in Fig. 1

**Fig. 5:**
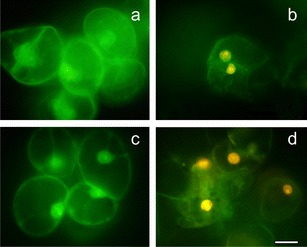
Micrographs of living and dying tomato cells detected by AO/EB staining. *Green nuclei* of living control cells in Perkoz (**a**) and Corino (**c**) cultures, *yellow nuclei* of cells dying via vacuolar type of death (**b**) in *B. cinerea*—inoculated (12 hpi) Perkoz cell culture, *orange*-*bright*-*red* nuclei of necrotic cells (**d**) in *B. cinerea*—inoculated (12 hpi) Corindo cell culture. *Bar*: 20 μm is applied to (**a**–**d**)

Evans blue staining and fluorescence analyses under the fluorescence microscopy after successive addition of fluorochromes AO/EB revealed that *B. cinerea* infection development proceeded slower in tomato cell cultures of cv. Perkoz than in cv. Corindo. Conidia of the pathogen started to germinate in both cell cultures at 6 hpi, but at that time, 38 % of conidia germinated in Corindo and 22 % in Perkoz cell cultures, respectively (Fig. 1). About 45 % of conidia germinated and formed shorter, swollen germination tubes with yellow-orange nuclei in Perkoz cell cultures (Figs. 1, 2, and 3a) which suggested that cells of the fungus died in vacuolar type of death. At that time, 70 % of conidia germinated and formed long and slender germination tubes with green, alive nuclei in Corindo cell cultures 12 hpi (Figs. 1, 2, and 3b). Infection proceeded and about 80 % of conidia germinated in both studied cell cultures at 24 hpi. Parallel to infection development, the viability of tomato cells was reduced from about 88 % in healthy cell cultures to 74 and 64 %, respectively, in Perkoz and Corindo ones at 12 hpi (Fig. 4). Round, uniformly green stained, large central nuclei were observed among alive cells of both cultures (Fig. 5a, c). Green-yellow and yellow nuclei with slightly condensed chromatin dominated among dying cells, indicating that these cells underwent early stages of cell death in the pathogen inoculated Perkoz cell cultures at that time (Fig. 5b). Cell death of those cells was accompanied by the characteristics that are proposed to be associated with vacuolar plant cell death. Partial disappearance of nuclei and nuclei with dark orange chromatin were visible in Corindo cells, suggesting necrotic type of cell death (Fig. 5d). About 50 % of the cells were dead in both cell cultures at 24 hpi; in both cultures, no viable cells were observed 48 h after challenge (Fig. 4).

### Content and cellular localization of ROS and NO generation during tomato cell cultures—*B. cinerea* interaction

**Fig. 6:**
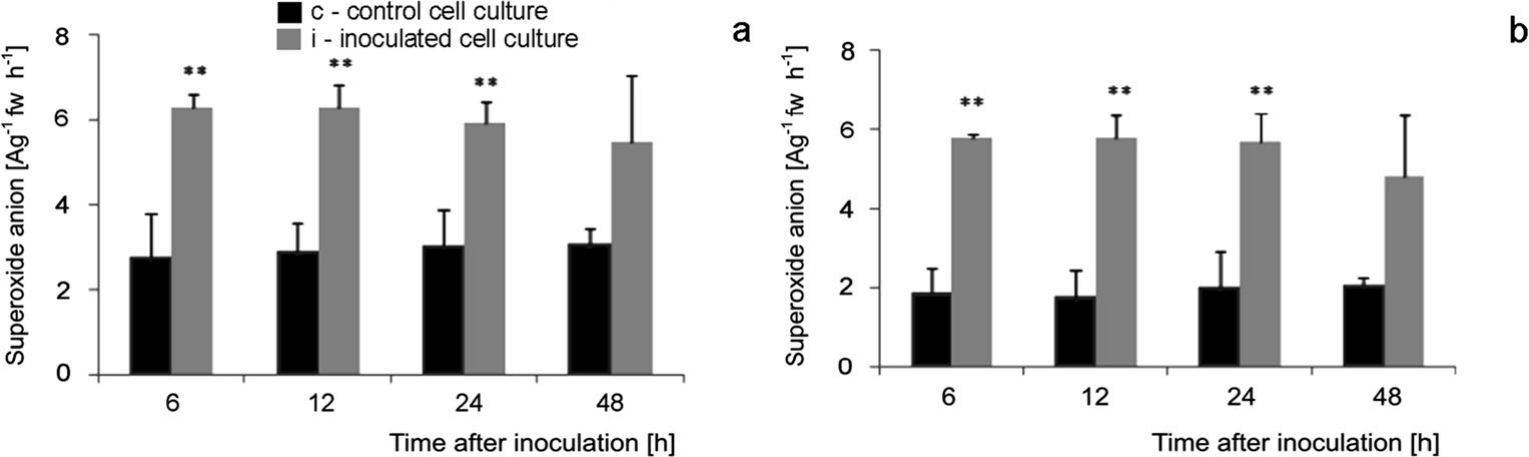
Superoxide anion concentration in Perkoz (**a**) and Corindo (**b**) cell cultures. Data and statistic as in Fig. 1; **P < 0.01 indicate values that differ significantly from the control

**Fig. 7:**
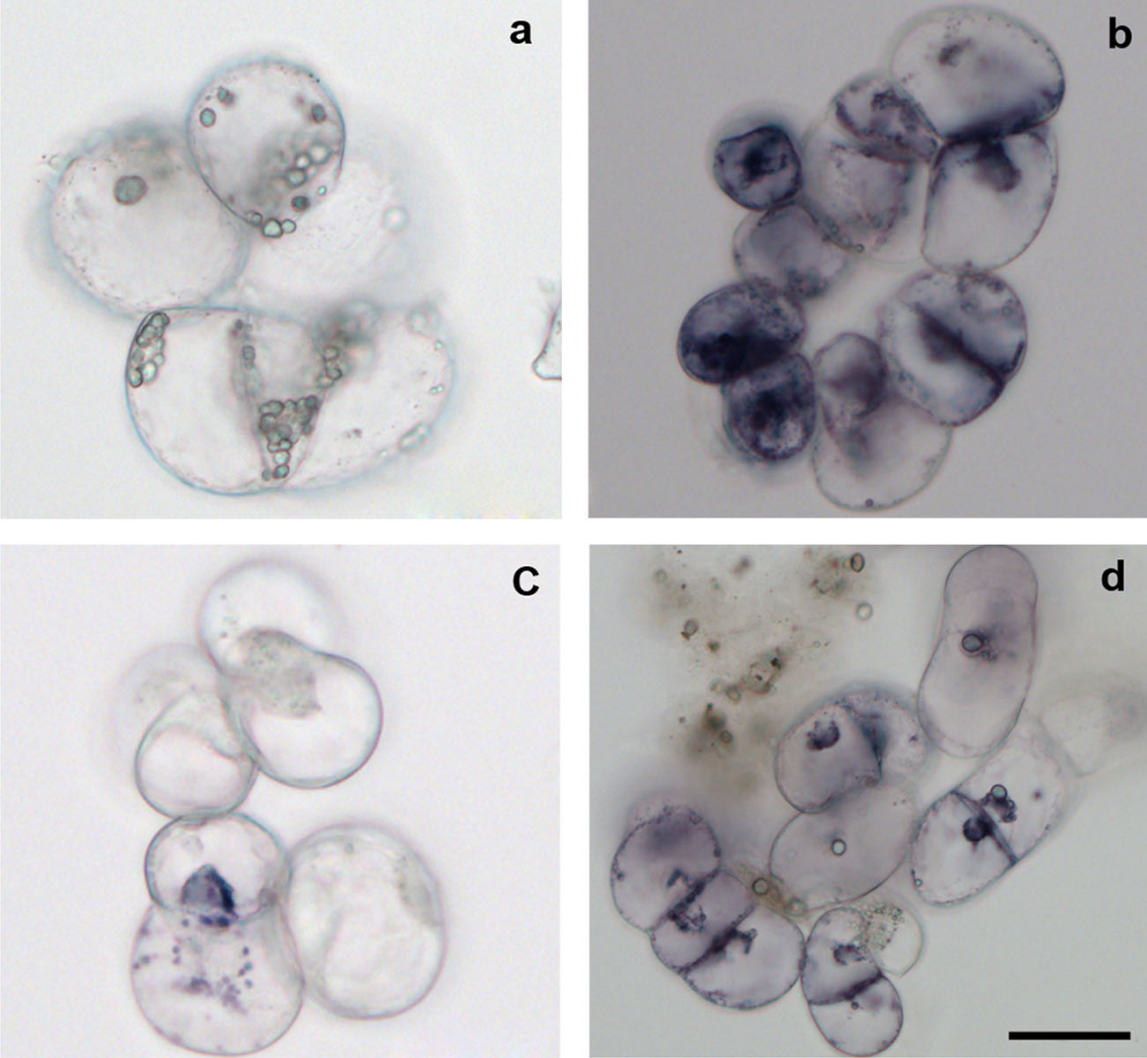
Superoxide anion detected by NBT staining in Perkoz control (**a**) and *B. cinerea* inoculated (**b**) cell cultures and in Corindo control (**c**) and *B. cinerea* inoculated (**d**) cell cultures at 12 hpi. *Bar*: 20 μm is applied to (**a**–**d**)

**Fig. 8:**
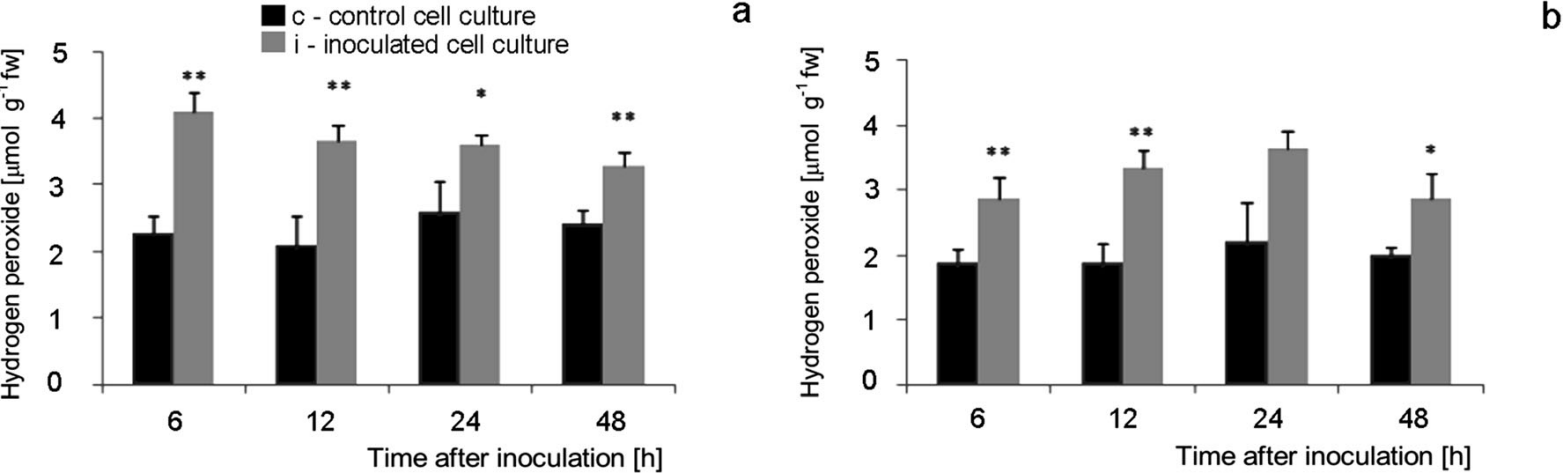
Hydrogen peroxide concentration in Perkoz (**a**) and Corindo (**b**) cell cultures. Data and statistic as in Fig. 1; *P < 0.05 and **P < 0.01 indicate values that differ significantly from the control

**Fig. 9:**
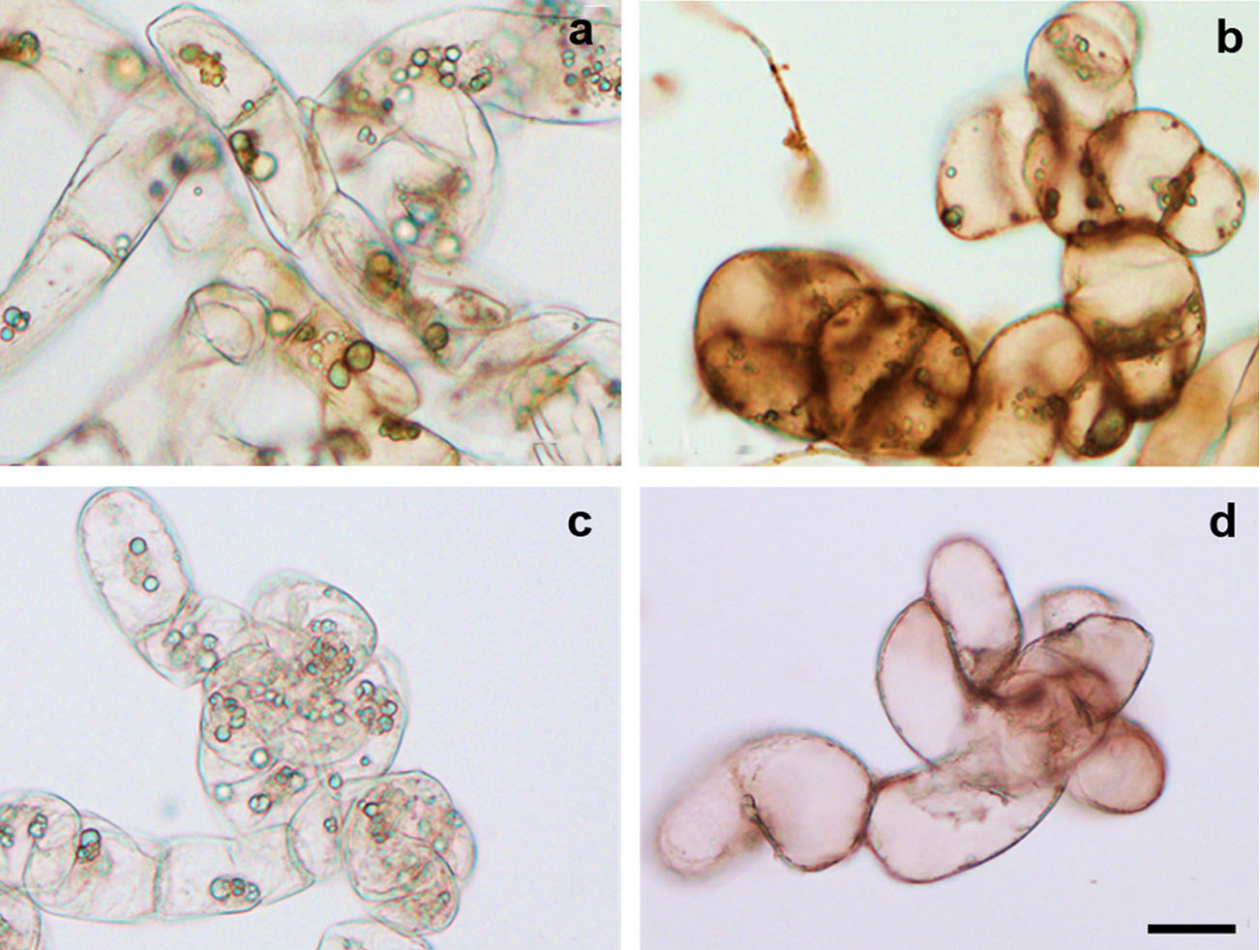
Hydrogen peroxide detected by DAB staining in Perkoz control (**a**) and *B.cinerea* inoculated (**b**) cell cultures and in Corindo control (**c**) and *B. cinerea* inoculated (**d**) cell cultures at 12 hpi. *Bar*: 20 μm is applied to (**a**–**d**)

**Fig 10:**
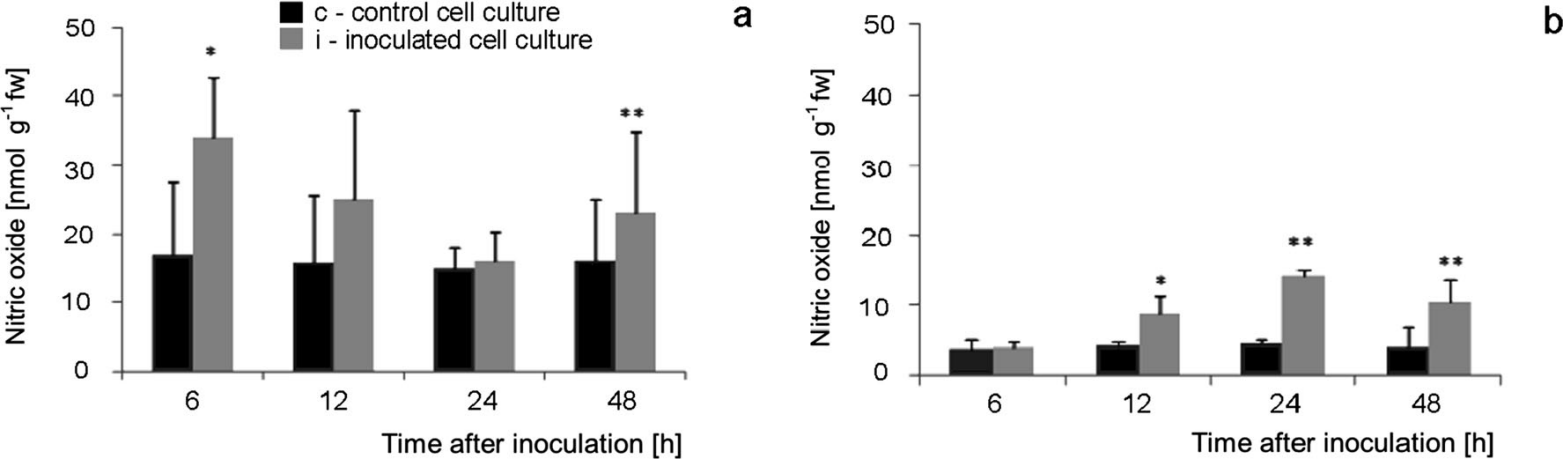
Nitric oxide concentration in Perkoz (**a**) and Corindo (**b**) cell cultures. Data and statistic as in Fig. 1; *P < 0.05 and **P < 0.01 indicate values that differ significantly from the control

NBT and DAB staining used for and microscopy detections of O_2_^.-^ and H_2_O_2_, respectively, as well as NO detection by CLSM system were carried out following biochemical, quantitative measurements of ROS, and NO. The data indicated that constitutive level of O_2_^.-^ was about two times higher, and H_2_O_2_ level was slightly higher in Perkoz than in Corindo (Figs. 6a,b, 7a–d, 8a,b and 9a-d). Constitutive, pre-inoculation NO concentration in Perkoz cell cultures was about 15 nmol g^−1^fw, about three-fold greater than in Corindo ones (about 5 nmol gfw^−1^) (Figs. 10a, b). Both tomato cell cultures reacted to inoculation with *B. cinerea* with enhanced synthesis of O_2_^.-^, H_2_O_2_ and NO. In Perkoz, O_2_^.-^generation measured as NBT reducing activity was about two times higher than in non-inoculated cultures at 6 hpi; this difference persisted throughout the experiment (Figs. 6a and 7a, b). Similarly, threefold increase in O_2_^.-^ concentration was observed in Corindo cell culture as a result of the pathogen inoculation (Fig. 6b). H_2_O_2_ concentration in the inoculated Perkoz cell cultures reached the maximal level 4 μmol g^−1^fw, about two times higher than in the control, as early as 6 hpi, and it persisted for up to 24 hpi (Fig. 8a, and 9a, b). The H_2_O_2_ concentration in the inoculated Corindo cell cultures increased to 3 μmol g^−1^fw at 6 and to 3.5 μmol g^−1^fw at 12 and 24 hpi, respectively (Figs. 8b, and 9c, d).

**Fig. 11:**
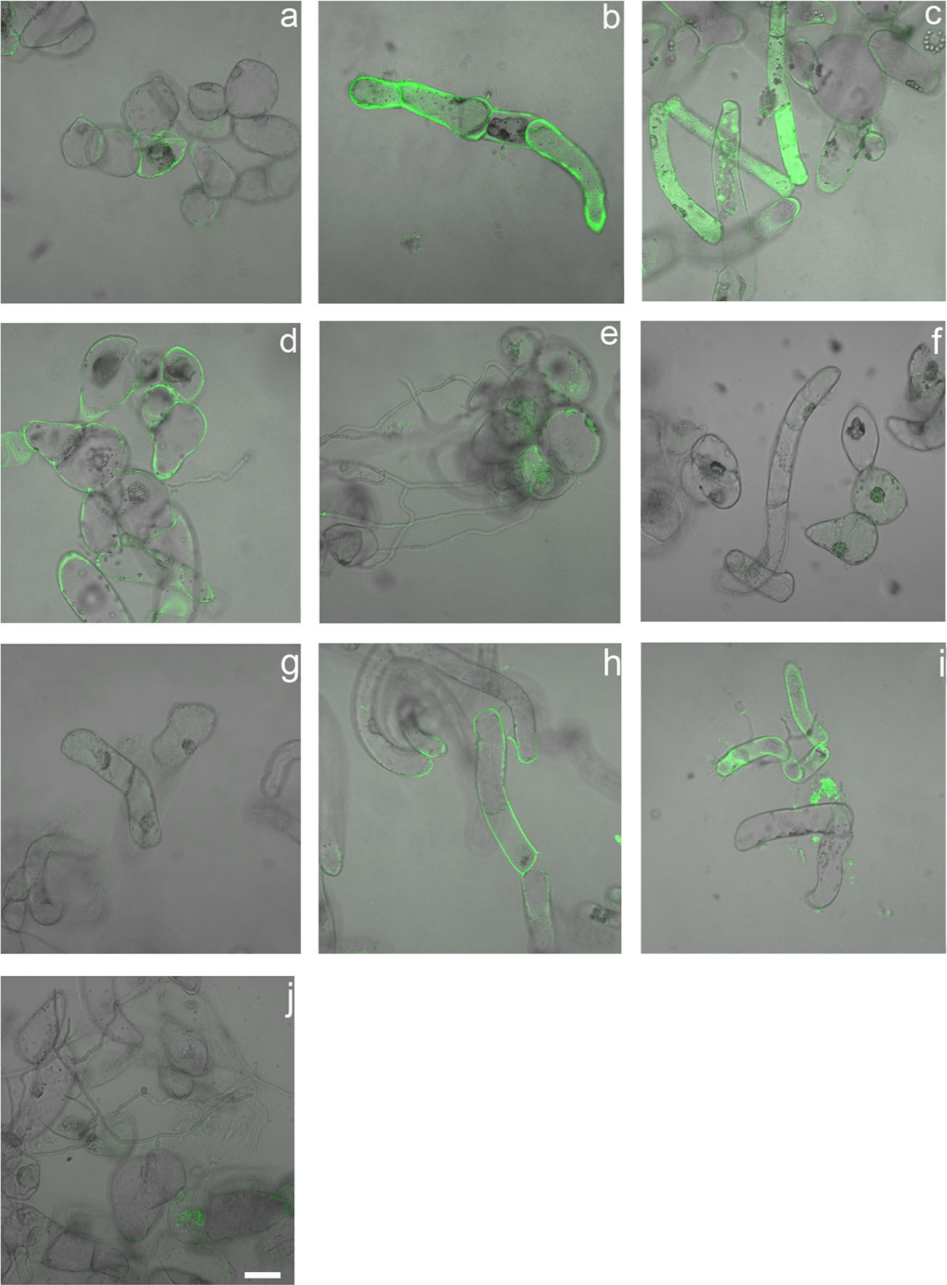
Nitric oxide detected by DAF-DA staining in Perkoz control (**a**) and *B. cinerea* inoculated cell cultures at 6 (**b**), 12 (**c**), 24 (**d**), and 48 (**e**) hpi as well as in Corindo control (**f**) and *B. cinerea* inoculated cell cultures at 6 (**g**), 12 (**h**), 24 (**i**), and 48 (**j**) hpi. *Bar*: 50 μm is applied to (**a**–**j**)

Nitric oxide concentration in Perkoz cell cultures reached 33 nmol gfw^−1^, the value two times higher than in the control at 6 hpi (Fig. 10a). After this significant increase, the decreasing trend in the level of NO to 25 nmol g^−1^fw at 12 hpi was observed in the inoculated cultures, than the next increase in NO generation at 48 hpi to 23 nmol g^−1^fw was noticed while NO level 15 nmol g^−1^fw was observed in the non-inoculated cultures. Similar trend of changes in NO concentrations was observed in Corindo cell cultures inoculated with the pathogen (Fig. 10b). Nitric oxide concentration in those cultures first increased 12 hpi to 10 nmol g^−1^fw, and then the increases were observed at 24 hpi to 16 nmol g^−1^fw and 48 hpi when NO concentration in the inoculated cultures reached 12 nmol g^−1^fw (Fig. 10b). Similar differences in NO content in the tomato cells were observed when the molecule was evaluated by CLSM (Fig. 11a–j)

### SNO content during tomato cell cultures—*B. cinerea* interaction

**Fig. 12:**
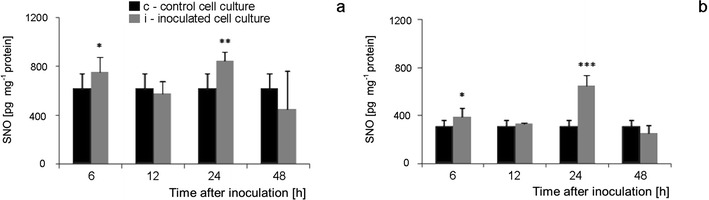
SNO concentration in Perkoz (**a**) and Corindo (**b**) cell cultures. Data and statistic as in Fig. 1;*P < 0.05, **P < 0.01, and ***P < 0.001 indicate values that differ significantly from the control

Constitutive SNO level in Perkoz cell cultures reached 600 pg mg^−1^ protein and was about two times higher than in Corindo (Figs. 12a, b). SNO concentration in Perkoz cultures markedly increased to about 700 pg mg^−1^ protein at 6 hpi; the next, similar increase in SNO concentration was observed in those cultures 24 hpi. SNO concentration in Corindo cell cultures increased only at 24 hpi when it reached the maximal level 600 pg mg^−1^ protein.

### Lipid peroxidation during tomato cell cultures—*B. cinerea* interaction

**Fig. 13:**
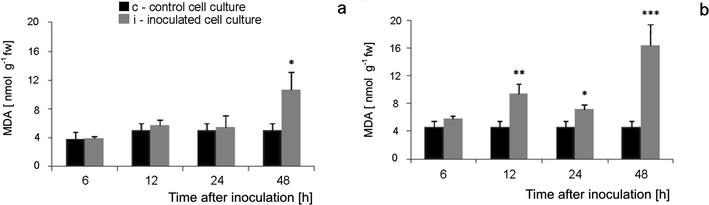
MDA concentration in Perkoz (**a**) and Corindo (**b**) cell cultures. Data and statistic as in Fig. 1; *P < 0.05, **P < 0.01, and ***P < 0.001 indicate values that differ significantly from the control

The extent of cell damage caused by reactive oxygen species and nitric oxide related to cell culture response to pathogen infection was estimated by the measurement of malonodialdehyde (MDA) content, the product of lipids peroxidation. In Corindo cell cultures inoculated with the pathogen, MDA content started to increase already 6 hpi, was significantly higher as compared to the control at 12 and 24 hpi and reached the highest, strongly significant level at 48 hpi (Fig. 13b). MDA concentration in Perkoz cell cultures increased less significantly only at the last stage of interaction (48 hpi) when there were no viable tomato cells in the culture (Fig. 13a).

### GSNOR activity during tomato cell cultures—*B. cinerea* interaction

**Fig. 14:**
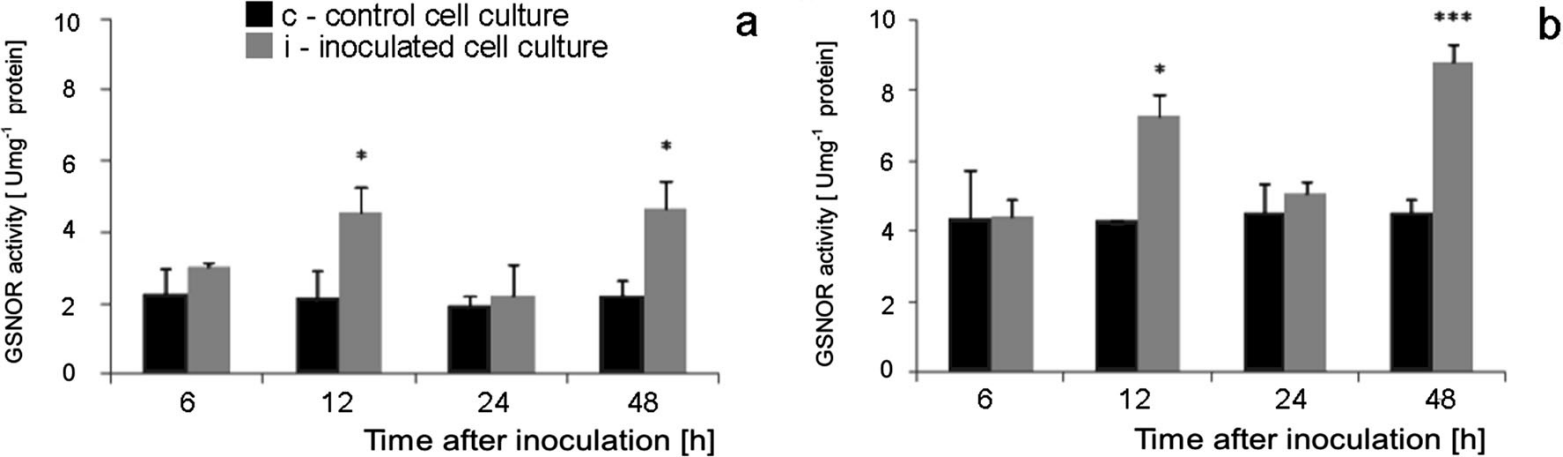
GSNOR activity in Perkoz (**a**) and Corindo (**b**) cell cultures. Data and statistic as in Fig. 1; *P < 0.05 and ***P < 0.001 indicate values that differ significantly from the control

The constitutive activity level of *S*-nitrosoglutathione reductase (GSNOR) was twice higher in Corindo cell cultures as compared with Perkoz ones (Figs. 14a, b). In Perkoz cell cultures, the enzyme activities started to increase already 6 hpi and reached the maximal, above two times higher level of activity as compared to the control at 12 hpi. The next, similar increase in the enzyme activity was observed at 48 hpi. In Corindo cell cultures inoculated with the pathogen, the increases in GSNOR activity were observed at 12 and 48 hpi, the latter being greater, twice higher than the control.

## Discussion

The present study investigated the *B. cinerea* infection development in tomato cell cultures of cv. Perkoz and cv. Corindo, less and more susceptible to the pathogen, respectively. Simultaneously, the effects of the pathogen on dying tomato cells and production of ROS, NO, SNO, MDA content, and changes in GSNOR activity as defense responses in them were studied.

The results indicate that the outcome of tomato cells—*B. cinerea* interaction, resistance, or susceptibility, strongly depended on the balance between cell death and survival; the type of cell death seemed to be of special importance. *B. cinerea* infection development was different in the studied cell cultures, especially at the beginning of the interaction. Conidia of the pathogen germinated earlier and formed alive, long, and slender germination tubes in the more susceptible cell cultures whereas in the less susceptible ones, they germinated slowly and formed shorter, swollen germination tubes consisting of cells with yellow-orange nuclei, which suggested that they died in vacuolar type of death (Byczkowska et al. 2013).

Simultaneously the tomato cells died in two different ways in the studied cultures: losses of nuclei or red nuclei indicating necrotic type of death dominated in Corindo cells; in plant cell cultures, this type of death is also described as apoptosis-like (Love et al. 2008). In Perkoz cells, early stages of PCD with different to necrosis symptoms, i.e., green-yellow and yellow nuclei with condensing chromatin were visible, indicating the type of programmed death described in literature (Byczkowska et al. 2013) as vacuolar.

These observations are in agreement with the results of other studies indicating that cell death program leading to rapid necrosis facilitates plant infection by necrotrophic pathogens such as *B. cinerea* (Dickman et al. 2001; Govrin and Levine 2000; Kars et al. 2005; Perchepied et al. 2010; van Baarlen et al. 2007).

Study on peroxidation of unsaturated lipids in biological membranes, the most prominent symptom of cell damage and marker of oxidative stress, confirmed that after the pathogen inoculation cells in Perkoz culture survived better and their viability was higher than in Corindo culture during the first stages (6–12 hpi) of interaction. The higher increases in lipid peroxidation measured by MDA concentration were demonstrated in Corindo culture, and they persisted throughout the experiment. MDA content increased also in the less susceptible culture but only at the later stages of interaction when both kinds of cell cultures were strongly infected with *B. cinerea* because of very favorable conditions for the pathogen development, e.g., due to presence of sugar and macro- and micronutrients in the culture medium.

Plants develop various strategies to defend themselves against different microbial pathogens. Death of plant cells and necrosis during HR, a well documented type of PCD in plants, characterized by rapid death of cells surrounding infection site, is one of common and effective plant defense strategies especially against biotrophic pathogens feeding on living host tissues (Choi et al. 2013; Iakimova et al. 2013). Cell death during HR may be advantageous for necrotrophs whose success in plant tissue colonization depends on the ability to kill host cells. In different plants, the relationship between cell death in HR and *B. cinerea* invasion is controversial. Research concerning *Arabidopsis thaliana*, tobacco, and other plants suggest that *B. cinerea* may induce HR cell death to achieve pathogenicity (Dickman et al. 2001; Govrin et al. 2006; van Baarlen et al. 2007; Kars et al. 2005). On the other hand, there are strong suggestions that cell death in HR may be also responsible for plant resistance to necrotrophs, depending on its timing and extent (Kunz et al. 2006; Asselbergh et al. 2007).

NO and ROS are important signaling molecules that are rapidly generated in plants after challenge with pathogens during nitrosative and oxidative burst. These compounds have been strongly implicated in controlling plant resistance and susceptibility. Their cooperation in the process of plant cell death was strongly suggested; the balance between intracellular NO and ROS levels is of special importance (Clarke et al. 2000; Bright et al. 2006; Zaninotto et al. 2006; Yun et al. 2011). Although the roles of ROS and NO and their contribution to plant resistance/susceptibility to pathogens was intensively studied, many details still need elucidation.

*B. cinerea* is a typical necrotroph, and despite extensive research on the biochemical bases of plant resistance mechanisms against this pathogen, the role of ROS and their cooperation with NO in this process are still controversial and far from clear. It was reported that enhanced ROS generation was found to accompany an infection caused by necrotrophs and even served as their weapon (Tiedemann 1997; Govrin and Levine 2000). On the other hand, some studies suggested positive effect of ROS and NO on plant resistance to necrotrophic pathogens like *B. cinerea* (Asai and Yoshioka 2009; Aziz et al. 2004; Floryszak-Wieczorek et al. 2007; Małolepsza and Urbanek 2002; Rasul et al. 2012).

To explain the role of ROS and NO in tomato—*B. cinerea* interaction two cultivars, more and less susceptible, were used to investigate the O_2_^.-^, H_2_O_2_, NO, and SNO generation using biochemical and in situ staining methods. As increased production of ROS and NO is a general reaction of plant/cells to different stimuli and may be triggered by different factors such as mechanical stress during biochemical procedures or changing environmental conditions, to eliminate the above, the biochemical study was complemented by in vivo ROS and NO imaging using confocal microscope technique allowing precise localization and determination of the role of O_2_^.-^, H_2_O_2_, and NO production/accumulation during tomato cell—*B. cinerea* interaction. The data on NO and ROS distribution acquired by confocal microscopy were generally consistent with the results obtained by the biochemical study. The tomato cell cultures reacted to inoculation with the pathogen with enhanced synthesis of O_2_^.-^and H_2_O_2_, in parallel burst of NO and some increase in SNO synthesis, were also noted. These phenomena were observed earlier and were more intensive in Perkoz, the less susceptible cell cultures, than in Corindo, the more susceptible ones. It is worth noting that constitutive, pre-inoculation NO and SNO concentrations in Perkoz cell cultures were about three and two times higher, respectively, than those in Corindo cultures. The results are in line with those presented by Rasul et al. (2012) that showed that susceptibility of *A. thaliana* plants to *B. cinerea* was related to reduce ability to synthesize NO. A specific role of NO in *A. thaliana* defense activation against another necrotrophic fungal pathogen, *Sclerotinia sclerotiorum* was also proved by Perchepied et al. (2010). The correlation between NO production in uninoculated tomato leaves and the level of tomato genotype resistance to biotrophic pathogen *Oidium neolycopersici* was reported by Piterkova et al. (2009).

Constitutive, pre-inoculation ROS concentration was also higher in Perkoz cell cultures as compared to Corindo ones. Thus both, higher constitutive levels of NO, SNO, and ROS as well as the capacity to produce these compounds faster and more effectively in the less susceptible tomato cells accompanied by slower *B. cinerea* infection development indicate that tomato defense reactions against the pathogen are evident at the cellular level. Moreover, it was observed that the studied cultivars displayed opposite behaviors against fungal infection and that ROS and specially NO and SNO crucially participated in biochemical bases of tomato cells resistance to *B. cinerea*. The molecules may act as direct antimicrobial agents delaying *B. cinerea* conidia germination; moreover, their role as signal molecules for further defense responses is also important (Hong et al. 2008; Perchepied et al. 2010; Romero-Puertas and Delledonne 2003; Wang and Higgins 2005).

Recently, much attention has been paid to the *S*-nitrosylation which is a signal modification.

*S*-nitrosothiols (SNO) also may function as an intracellular reservoir of NO (Chaki et al. 2009). Differences between the concentrations of these compounds in the studied tomato cell cultures before and after inoculation with *B. cinerea* indicated that SNO might also be an important component of signaling pathways triggered in response to *B. cinerea* attack as suggested by Wang et al. (2009) and Hong et al. (2008).

In animal cells, *S*-nitrosoglutathione reductase is a key enzyme responsible for maintaining the homeostasis of *S*-nitrosothiols. Recently, there have been reports suggesting that this enzyme may play a similar role in plant cells (Lin et al. 2012; Malik et al. 2011). Benhar et al. (2009) showed that *S*-nitrosoglutathione reductase protected plant cells and affected defense mechanisms in plant-pathogen interactions, also Gupta et al. (2011) indicated that modulation of GSNOR activity in plants had a significant impact on the ability of plants to defend themselves against pathogens.

Two times higher constitutive level of GSNOR activity in Corindo cell cultures as compared to Perkoz ones and increase in the activity of this enzyme in response to the pathogen are conducive to reducing the concentration of SNO in tomato cell cultures and may be related to the susceptibility to *B. cinerea*. Similarly, to our results, transgenic *A. thaliana* plants with reduced amounts of GSNOR and concomitant increased intracellular SNO level have enhanced resistance against *Peronospora parasitica* (Restérucci et al. 2007). Contrary to that, Feechan et al. (2005) postulated that the reduction of *S*-nitrosothiols concentration as a result of increased GSNOR activity in *A. thaliana* was related to protection against microbial infection.

In conclusion, we indicated that *B. cinerea* inoculation of two tomato cell cultures differing in susceptibility to the pathogen induced two different types of cell death. The type of cell death accompanied by the characteristics that are proposed to be associated with vacuolar plant cell death dominated in tomato cells less susceptible to the pathogen whereas necrotic/apoptotic-like type of death dominated in more susceptible ones at early stages of interaction.

Constitutive level and speed of NO, SNO, and ROS generation after *B. cinerea* inoculation appear to limit plant cell death and development of necrosis. Moreover, those plant defense molecules might target *B. cinerea* cell death machinery and slow down the pathogen infection development. The results indicate that NO, SNO, and ROS are important, early signals during the tomato cells—*B. cinerea* interaction and form a part of molecular mechanism of tomato resistance to the pathogen.
